# Supplementary material for: Development and Validation of a Questionnaire of the Perioperative Nursing Competencies in Patient Safety
Source: Int J Environ Res Public Health. 2022 Feb 23;19(5):2584. doi: 10.3390/ijerph19052584 (PMC8909926; doi:10.3390/ijerph19052584)
Supplement: Supplementary file 1 [file ijerph-19-02584-s001.zip › ijerph-1571214-SM.pdf]

Supplementary Materials:

Table S1. Detailed information of the experts.

Name and surname of the expert perioperative nurses	Profession	Institution
1. Rosa Gutiérrez Romero.	Perioperative Nurse	Consorci Sanitari de Terrassa (CST)
2. Raquel Navarro Franco	Perioperative Nurse	University Hospital Mútua de Terrassa
3. MªJosé Arch Pintó	Perioperative Nurse	Consorci Sanitari de Terrassa (CST)
4. María Castilla de la Serna	Perioperative Nurse	University Hospital Puerta de Hierro Majadahonda
5. Catalina Méndez Martínez	Perioperative Nurse	Consorci Sanitari de Terrassa (CST)
6. Maria Ascensión Martínez Alonso	Perioperative Nurse	Consorci Sanitari de Terrassa (CST)
7. María José Funes Meseguer	Perioperative Nurse	Reina Sofía Hospital (Murcia)
8. Rosa María Romero Cobo	Perioperative Nurse	Hospital Universitari Mútua de Terrassa
9. Maite Hernández Galvan	Perioperative Nurse	Consorci Sanitari de Terrassa (CST)
10. Sandra Monné Collado	Perioperative Nurse	Consorci Sanitari de Terrassa (CST)
11. Esther Espuñes Mestres	Perioperative Nurse	ASEPEYO Sant Cugat del Vallès Hospital Escola Universitària Infermeria Gimbernat
12. Lorenza Marmol Reyes	Perioperative Nurse	Esperit Sant Hospital Foundation
13. Noelia Gómez Delgado	Perioperative Nurse	Hospital Universitari Vall d'Hebron
14. Pilar Eguiluz Fernandez	Perioperative Nurse	Hospital Universitario Puerta de Hierro Majadahonda
15. Miracle Flores Gonzalez	Perioperative Nurse	Hospital Sant Bernabé
16. Paqui Roldán Delgado	Perioperative Nurse	Fundació Hospital Esperit Sant
17. Jenny Martínez Alvarez	Perioperative Nurse	Hospital Universitario de la Paz (Madrid)
18. MªJosé Lorca	Perioperative Nurse	Hoapitla Reina Sofia (Murcia)
19. Mª Isabel Molina Iglesias	Perioperative Nurse	Hospital de Sevilla
20. Susana Herrera	Perioperative Nurse	Mc mutual
21. Dámaris Amoraga	Perioperative Nurse	Consorci Sanitari de Terrassa (CST)
22. Laura León Carrillo	Perioperative Nurse	Hospital Universitari Mútua de Terrassa
23. Emma Batllé Meler	Perioperative Nurse	Hospital Universitari Mútua de Terrassa
24. Rafel Llorens Ortega	Perioperative Nurse	Escola Universitària d'Infermeria i Teràpia Ocupacional de Terrassa
25. Fátima Sabrina Peters Gutiérrez	Perioperative Nurse	Hospiatl Universitario Puerta de Hierro Majadahonda
**Name and surname experts in clinical safety**	**Profession/Institution**	**Current work activity**
1. Cristina Sagrera Felip	Nurse Hospital Universitari General de Catalunya, Grupo Quironsalud	Person of reference for Patient Safety
2. Vicente Santana López	Nurse Agencia de Calidad Sanitaria de Andalucía	Person of reference for Business Development and Quality 3.00
3. María Cruz Martín Delgado	Doctor	Director of the ISDE Health Area

	Intensive Care Unit.<br>Hospital Universitario de Torrejón	President of FIDISP (Foundation for research, teaching and innovation in patient safety)
4. Yolanda Agra Varela	Doctor<br>Ministerio de Sanidad, Servicios Sociales e Igualdad Unidad Seguridad del paciente	Quality Assistant
5. Montserrat Comellas Oliva	Nurse<br><br>Escola Universitaria de Infermeria i Terapia Ocupacional de Terrassa (EUIT)	Director<br><br>Quality and safety expert
6. Adelina García Matarín	Nurse<br><br>Escola Universitaria de Infermeria i Terapia Ocupacional de Terrassa (EUIT)<br><br>Miembro de la fundación FIDISP (Fundación por la investigación, docencia e innovación en seguridad del paciente)	Research professor<br><br>Safety expert
7. Santiago Tomas Vecina	Doctor<br><br>Badalona Serveis Assistencials<br><br>Director of the la FIDISP fundation	Head of the Quality and Patient Safety Unit
8. Rosa Maria Muñoz Albarracin	Nurse<br><br>Hospital de la Santa Creu i Sant Pau de Barcelona	Head of Nursing of the Surgical and Ophthalmology Area<br><br>President of the Patient Safety Commission
9. Pilar Hilarion Madariaga	Nurse<br><br>Avedis Donabedian University Institute - UAB	Director of the Avedis Donabedian University Institute
10. Nuria Freixas Sala	Nurse<br><br>Hospital Universitari Mútua de Terrassa	Development and training area in patient safety
11. Adriana Caterina de Souza	Nurse<br><br>Universidad Católica San Antonio de Murcia	Public health<br>Patient Safety Area<br><br>Professor and researcher
12. Joaquim Bañeres Amella	Doctor	Director of the Avedis Donabedian University

	Avedis Donabedian University Institute – UAB	Institute.<br><br>Collaborator of the Ministry of Health, various Health Departments, WHO and numerous national and international governmental and non-governmental organizations.
13. María Encarna Martínez Hernández	Nurse<br><br>Consorcio Hospital General Universitario de Valencia.<br><br>Escuela Valenciana de Estudios para la Salud (EVES)	Perioperative Nurse<br>Management processes and procedures<br><br>Professor and researcher

Table S2. Panel of experts consensus according to sub-competencies. Round 2 and 3.

Subcompetencie	ROUND 2		ROUND 3	
	MEAN(SD)	CVI(SD)	MEAN(SD)	CVI(SD)
**C1**				
C1EC1	4.80(0.18)	0.98(0.03)	4.85(0.16)	0.98(0.03)
C1EC2	4.5(0.19)	0.88(0.08)	4.46(0.23)	0.86(0.07)
**C2**				
C2EC1	4.76(0.15)	0.95(0.04)	4.85(0.13)	0.99(0.03)
C2EC2	4.75(0.08)	0.95(0.03)	4.73(0.11)	0.96(0.04)
C2EC3	4.61(0.25)	0.94(0.06)	4.67(0.35)	0.94(0.10)
C2EC4	4.77(0.20)	0.95(0.05)	4.78(0.22)	0.97(0.04)
C2EC5	4.89(0.10)	0.99(0.02)	4.91(0.10)	0.99(0.02)
C2EC6	4.74(0.05)	0.95(0.02)	4.86(0.05)	0.97(0.02)
C2EC7	4.82(0.12)	0.98(0.02)	4.83(0.14)	0.97(0.03)
C2EC8	4.75(0.13)	0.97(0.03)	4.80(0.10)	0.99(0.01)
**C3**				
C3EC1	4.57(0.07)	0.95(0.01)	4.63(0.07)	0.95(0.02)
C3EC2	4.55(0.07)	0.93(0.01)	4.65(0.06)	0.96(0.02)
C3EC3	4.40(0.18)	0.89(0.05)	4.43(0.19)	0.89(0.06)
**C4**				
C4EC1	4.16(0.27)	0.81(0.08)	4.27(0.28)	0.83(0.10)
C4EC2	4.40(0.20)	0.88(0.05)	4.46(0.16)	0.91(0.06)
C4EC3	4.63(0.28)	0.93(0.08)	4.61(0.24)	0.93(0.08)
C4EC4	4.56(0.19)	0.93(0.05)	4.52(0.18)	0.92(0.06)

C1: Ethical and legal practice; C2: Perioperatory care; C3: Communication; C4: Culture of Safety. C1EC1: Safety Standards; C1EC2: Surgical Checklist; C2EC1: Anaesthesiology Nurse; C2EC2: Safe placement of the patient; C2EC3: Surgical Thermoregulation; C2EC4: Circulating Nurse; C2EC5: Scrub Nurse; C2EC6: Safe use of the electric scalpel; C2EC7: Postoperative Nurse; C2EC8: Postoperative pain; C3EC1: Efficient communication with the user; C3EC2: Communication and teamwork; C3EC3: Leadership; C4EC1: Professional culture of safety; C4EC2: Institutional culture of safety; C4EC3: Errors; C4EC4: Knowledge.

Table S3. CUCEQS© scores according to perioperative nursing.

Competency/ Sub-competency	Numberof items	Final score	Maximum score by competency according to Role
**Competency 1: Legislation, ethics, andprofessional orientation**	11	44	44
Comply with safety standards	8	32	
Application of the Surgical Checklist	3	12	
**Competency 2: Perioperative nursingcare**			Anaesthesia Nurse: 144<br>Circulating Nurse: 152<br>Scrub Nurse: 88<br>Postoperative Nurse: 120
Anaesthesia Nurse:	12	48	
Circulating Nurse or Anaesthesia Nurse-Patient positioning	6	24	
Circulating Nurse or Anaesthesia Nurse-Surgical thermoregulation	5	20	
Circulating Nurse	14	56	
Scrub Nurse	16	64	
Scrub Nurse, Circulating Nurse, Anaesthesia Nurse - Electric scalpel	6	24	
Postoperative Nurse	23	92	
Postoperative Nurse , Circulating Nurse,Anaesthesia Nurse- Postoperative Pain	7	28	
**Competency 3: Effective interpersonalrelationship with the patient and surgical team**	22	88	88
Effective communication	7	28	
Leadership	15	60	
**Competency 4: Safety Culture**	35	140	140
Develop Safety Culture focused on theprofessional	5	20	
Develop Safety Culture focused on the professional with respect to organizationor institution	6	24	
Notification of errors	14	56	
Develop scientific knowledge on theperioperative process	10	40	

Table S4. Comparison of the scores found in the test and retest.

	Test Median [Interquartilerange]	Retest Median [Interquartilerange]	p-value	N
**C1EC1**	30[27;32]	30[28;32]	0.338	109
**C1EC2**	10[7;12]	10[7;12]	0.566	109
**C1 TOTAL**	39[34;42]	40[36;42]	0.37	109
**C2EC1**	45[43;48]	45[42;47]	0.98	109
**C2EC2**	23[20;24]	23[20;24]	0.524	109
**C2EC3**	17[15;20]	18[15;20]	0.54	109
**C2EC4**	51[49;54]	52[49;54]	0.624	109
**C2EC5**	62[60;64]	62[60;64]	0.939	109
**C2EC6**	23[20;24]	23[21;24]	0.333	109
**C2EC7**	86[0;91]	86[3;91]	0.459	107
**C2EC8**	26[19;28]	26[20;28]	0.298	109
**C2 TOTAL**	298[218;317]	301[224;318.5]	0.48	107
**C3EC1**	25[22;27]	24[21;27]	0.933	109
**C3EC2**	24[21;28]	24[21;27]	0.656	109
**C3EC3**	47[43;54]	49[43.75;56]	0.303	108
**C3 TOTAL**	95[87;108]	98[87;110]	0.485	108
**C4EC1**	15[12;18]	15[12;19]	0.621	109
**C4EC2**	19[15;23]	19.5[16;23]	0.549	108
**C4EC3**	47[42;54]	48[43;53]	0.658	109
**C4EC4**	35[32;38]	35[32;38]	0.818	109
**C4 TOTAL**	114[103;129]	117[104;130]	0.568	108

C1: Ethical and legal practice; C2: Perioperatory care; C3: Communication; C4: Culture of Safety. C1EC1: Safety Standards; C1EC2: Surgical Checklist; C2EC1: Anaesthesiology Nurse; C2EC2: Safe placement of the patient; C2EC3: Surgical Thermoregulation; C2EC4: Circulating Nurse; C2EC5: Scrub Nurse; C2EC6: Safe use of the electric scalpel; C2EC7: Postoperative Nurse; C2EC8: Postoperative pain; C3EC1: Efficient communication with the user; C3EC2: Communication and teamwork; C3EC3: Leadership; C4EC1: Professional culture of safety; C4EC2: Institutional culture of safety; C4EC3: Errors; C4EC4: Knowledge.

Table S5. Intraclass correlation coefficient according to competency and sub-competency.

Sub-competency/Competency	Intraclass correlationcoefficient (ICC)
**C1EC1**	0.872 [95% CI: 0.826; 0.903]
**C1EC2**	0.817 [95% CI: 0.754; 0.862]
**C1 TOTAL**	0.881 [95% CI: 0.839; 0.91]
**C2EC1**	0.917 [95% CI: 0.887; 0.937]
**C2EC2**	0.935 [95% CI: 0.912; 0.951]
**C2EC3**	0.912 [95% CI: 0.881; 0.934]
**C2EC4**	0.867 [95% CI: 0.82; 0.9]
**C2EC5**	0.77 [95% CI: 0.691; 0.825]
**C2EC6**	0.875 [95% CI: 0.831; 0.906]
**C2EC7**	0.926 [95% CI: 0.899; 0.944]
**C2EC8**	0.805 [95% CI: 0.738; 0.852]
**C2 TOTAL**	0.887 [95% CI: 0.846; 0.915]
**C3EC1**	0.844 [95% CI: 0.79; 0.882]
**C3EC2**	0.887 [95% CI: 0.847; 0.915]
**C3EC3**	0.912 [95% CI: 0.881; 0.934]
**C3 TOTAL**	0.889 [95% CI: 0.85; 0.917]
**C4EC1**	0.833 [95% CI: 0.775; 0.874]
**C4EC2**	0.858 [95% CI: 0.807; 0.892]
**C4EC3**	0.853 [95% CI: 0.801; 0.889]
**C4EC4**	0.825 [95% CI: 0.764; 0.867]
**C4 TOTAL**	0.893 [95% CI: 0.854; 0.919]

C1: Ethical and legal practice; C2: Perioperatory care; C3: Communication; C4: Culture of Safety. C1EC1: Safety Standards; C1EC2: Surgical Checklist; C2EC1: Anaesthesiology Nurse; C2EC2: Safe placement of the patient; C2EC3: Surgical Thermoregulation; C2EC4: Circulating Nurse; C2EC5: Scrub Nurse; C2EC6: Safe use of the electric scalpel; C2EC7: Postoperative Nurse; C2EC8: Postoperative pain; C3EC1: Efficient communication with the user; C3EC2: Communication and teamwork; C3EC3: Leadership; C4EC1: Professional culture of safety; C4EC2: Institutional culture of safety; C4EC3: Errors; C4EC4: Knowledge.

Table S6. Scores according to competency and competency element of nurses who are experts in safety and perioperative nurses.

	Safety Experts	Perioperative Nurses	[ALL]	p-valor	N
	N=9	N=415	N=424		
**C1EC1**	32.0 [30.0;32.0]	30.0 [27.0;32.0]	30.0 [27.0;32.0]	0.073	424
**C1EC2**	12.0 [12.0;12.0]	10.0 [8.00;12.0]	10.0 [8.00;12.0]	0.005	424
**C1**	44.0 [42.0;44.0]	39.0 [35.0;42.0]	39.0 [35.0;42.0]	0.007	424
**C2EC1**	48.0 [0.00;48.0]	45.0 [41.0;47.0]	45.0 [41.0;47.0]	0.975	424
**C2EC2**	24.0 [0.00;24.0]	22.0 [20.0;24.0]	22.0 [20.0;24.0]	0.738	424
**C2EC3**	20.0 [0.00;20.0]	17.0 [15.0;20.0]	17.0 [15.0;20.0]	0.904	424
**C2EC4**	53.0 [0.00;56.0]	52.0 [48.0;54.0]	52.0 [48.0;54.0]	0.941	424
**C2C5**	62.0 [0.00;64.0]	62.0 [59.0;64.0]	62.0 [59.0;64.0]	0.521	424
**C2EC6**	23.0 [0.00;24.0]	23.0 [21.0;24.0]	23.0 [21.0;24.0]	0.405	424
**C2EC7**	92.0 [91.0;92.0]	85.0 [61.5;91.0]	85.5 [63.2;91.0]	0.027	424
**C2EC8**	28.0 [26.0;28.0]	26.0 [22.0;28.0]	26.0 [22.0;28.0]	0.223	424
**C2**	321 [92.0;328]	298 [226;316]	298 [225;316]	0.719	424
**C3EC1**	27.0 [25.0;28.0]	25.0 [22.0;27.0]	25.0 [22.0;27.0]	0.139	424
**C3EC2**	28.0 [23.0;28.0]	24.0 [21.0;27.0]	24.0 [21.0;27.0]	0.109	424
**C3EC3**	58.0 [49.0;60.0]	47.0 [44.0;53.0]	47.0 [44.0;53.2]	0.018	424
**C3**	113 [980;116]	95.0 [87.0;105]	96.0 [87.0;106]	0.027	424
**C4EC1**	20.0 [16-0;20.0]	15.0 [12.0;17.0]	15.0 [12.8;17.0]	0.001	424
**C4EC2**	24.0 [24.0;24.0]	18.0 [16.0;22.0]	18.0 [16.0;22.0]	<0.001	424
**C4EC3**	55.0 [54.0;56.0]	48.0 [43.0;53.0]	48.0 [43.0;53.0]	<0.001	424
**C4EC4**	38.0 [32.0;40.0]	35.0 [32.0;38.0]	35.0 [32.0;38.0]	0.147	424

C1: Ethical and legal practice; C2: Perioperatory care; C3: Communication; C4: Culture of Safety. C1EC1: Safety Standards; C1EC2: Surgical Checklist; C2EC1: Anaesthesiology Nurse; C2EC2: Safe placement of the patient; C2EC3: Surgical Thermoregulation; C2EC4: Circulating Nurse; C2EC5: Scrub Nurse; C2EC6: Safe use of the electric scalpel; C2EC7: Postoperative Nurse; C2EC8: Postoperative pain; C3EC1: Efficient communication with the user; C3EC2: Communication and teamwork; C3EC3: Leadership; C4EC1: Professional culture of safety; C4EC2: Institutional culture of safety; C4EC3: Errors; C4EC4: Knowledge.

Table S7. Scores according to competency and competency element of surgical master's nursing students who had received specific surgical patient safety training.

	Time 0				Time 1			
	**Safety training**	**No safety training**	**Total**	**p-value**	**Safety training**	**No safety training**	Total	p-value
	N=30	N=26	N=56		N=30	N=26	N=56	
**C1EC1**	32.0 [32.0;32.0]	32.0 [30.0;32.0]	32.0 [31.0;32.0]	0.022	32.0 [32.0;32.0]	32.0 [30.0;32.0]	32.0 [31.0;32.0]	0.022
**C1EC2**	12.0 [11.2;12.0]	12.0 [11.0;12.0]	12.0 [11.0;12.0]	0.577	12.0 [12.0;12.0]	12.0 [11.2;12.0]	12.0 [12.0;12.0]	0.173
**C1**	44.0 [43.0;44.0]	43.0 [42.0;44.0]	44.0 [42.0;44.0]	0.059	44.0 [44.0;44.0]	43.5 [41.0;44.0]	44.0 [43.0;44.0]	0.020
**C2EC1**	48.0 [46.0;48.0]	48.0 [45.2;48.0]	48.0 [46.0;48.0]	0.398	48.0 [48.0;48.0]	48.0 [47.0;48.0]	48.0 [47.0;48.0]	0.099
**C2EC2**	24.0 [23.0;24.0]	24.0 [22.0;24.0]	24.0 [23.0;24.0]	0.614	24.0 [24.0;24.0]	24.0 [22.2;24.0]	24.0 [24.0;24.0]	0.015
**C2EC3**	19.0 [18.2;20.0]	19.0 [18.0;20.0]	19.0 [18.0;20.0]	0.631	20.0 [19.2;20.0]	19.0 [18.0;20.0]	20.0 [18.8;20.0]	0.039
**C2EC4**	55.0 [53.0;56.0]	54.5 [50.2;56.0]	55.0 [52.0;56.0]	0.556	56.0 [54.0;56.0]	54.5 [51.2;56.0]	56.0 [54.0;56.0]	0.024
**C2EC5**	63.5 [63.0;64.0]	62.5 [61.0;64.0]	63.0 [62.0;64.0]	0.151	64.0 [63.0;64.0]	63.5 [59.5;64.0]	64.0 [62.0;64.0]	0.032
**C2EC6**	24.0 [24.0;24.0]	24.0 [24.0;24.0]	24.0 [24.0;24.0]	0.943	24.0 [24.0;24.0]	24.0 [23.2;24.0]	24.0 [24.0;24.0]	0.070
**C2EC7**	91.5 [90.0;92.0]	91.5 [89.2;92.0]	91.5 [90.0;92.0]	0.647	92.0 [92.0;92.0]	91.5 [87.0;92.0]	92.0 [90.0;92.0]	0.004
**C2EC8**	28.0 [28.0;28.0]	280 [26.0;28.0]	28.0 [27.0;28.0]	0.201	28.0 [28.0;28.0]	28.0 [26.0;28.0]	28.0 [27.0;28.0]	0.042
**C2**	322 [317;326]	317 [309;328]	320 [312;327]	0.337	327 [322;328]	318 [308;328]	326 [318;328]	0.008
**C3EC1**	28.0 [26.2;28.0]	27.0 [25.2;28.0]	28.0 [26.0;28.0]	0.213	28.0 [28.0;28.0]	27.0 [25.0;28.0]	28.0 [26.0;28.0]	0.003
**C3EC2**	28.0 [27.0;28.0]	28.0 [26.0;28.0]	28.0 [27.0;28.0]	0.837	28.0 [27.2;28.0]	27.5 [26.0;28.0]	28.0 [27.0;28.0]	0.016
**C3EC3**	58.5 [55.2;59.8]	58.0 [50.8;59.8]	58.0 [54.8;60.0]	0.483	60.0 [58.2;60.0]	58.5 [50.2;59.8]	59.0 [54.0;60.0]	0.027
**C3**	113 [110;115]	112 [104;115]	113 [107;115]	0.568	115 [113;116]	112 [100;115]	114 [108;116]	0.013
**C4EC1**	17.0 [16.0;20.0]	19.0 [16.0;20.0]	18.0 [16.0;20.0]	0.421	20.0 [17.0;20.0]	19.0 [16.0;20.0]	20 [16.8;20.0]	0..141
**C4EC2**	23.0 [20.0;24.0]	22.0 [18.0;24.0]	23.0 [19.0;24.0]	0.403	24.0 [23.0;24.0]	22.0 [20.0;24.0]	24.0 [20.8;24.0]	0.049
**C4EC3**	55.0 [52.0;56.0]	53.0 [50.0;56.0]	54.5 [50.0;56.0]	0.271	56.0 [55.0;56.0]	53.0 [51.2;56.0]	55.5 [53.0;56.0]	0.003
**C4EC4**	39.0 [35.2;40.0]	38.0 [36.2;39.0]	38.0 [35.8;40.0]	0.218	40.0 [38.2;40.0]	39.0 [35.5;40.0]	40.0 [37.0;40.0]	0.028
**C4**	132 [124;136]	128 [120;138]	131 [122;138]	0.469	137 [134;140]	128 [125;140]	136 [128;140]	0.018

C1: Ethical and legal practice; C2: Perioperatory care; C3: Communication; C4: Culture of Safety. C1EC1: Safety Standards; C1EC2: Surgical Checklist; C2EC1: Anaesthesiology Nurse; C2EC2: Safe placement of the patient; C2EC3: Surgical Thermoregulation; C2EC4: Circulating Nurse; C2EC5: Scrub Nurse; C2EC6: Safe use of the electric scalpel; C2EC7: Postoperative Nurse; C2EC8: Postoperative pain; C3EC1: Efficient communication with the user; C3EC2: Communication and teamwork; C3EC3: Leadership; C4EC1: Professional culture of safety; C4EC2: Institutional culture of safety; C4EC3: Errors; C4EC4: Knowledge.

Table S8. STROBE Statement—Checklist of items that should be included in reports of cohort studies.

	Item No	Recommendation	Page No
Title and abstract	1	(a) Indicate the study's design with a commonly used term in the title or the abstract	1
		(b) Provide in the abstract an informative and balanced summary of what was performed and what was found	1
		**Introduction**	
Background/rationale	2	Explain the scientific background and rationale for the investigation being reported	1,2
Objectives	3	State specific objectives, including any prespecified hypotheses	2
		**Methods**	
Study design	4	Present key elements of study design early in the paper	2
Setting	5	Describe the setting, locations, and relevant dates, including periods of recruitment, exposure, follow-up, and data collection	2,3
Participants	6	(a) Give the eligibility criteria, and the sources and methods of selection of participants. Describe methods of follow-up	2,3,6, Supp. Mat. S1 Table 2
		(b) For matched studies, give matching criteria and number of exposed and unexposed	
Variables	7	Clearly define all outcomes, exposures, predictors, potential confounders, and effect modifiers. Give diagnostic criteria, if applicable	3
Data sources/measurement	8*	For each variable of interest, give sources of data and details of methods of assessment (measurement). Describe comparability of assessment methods if there is more than one group	3,4
Bias	9	Describe any efforts to address potential sources of bias	5
Study size	10	Explain how the study size was arrived at	3
Quantitative variables	11	Explain how quantitative variables were handled in the analyses. If applicable, describe which groupings were chosen and why	3
Statistical methods	12	(a) Describe all statistical methods, including those used to control for confounding	3,4
		(b) Describe any methods used to examine subgroups and interactions	
		(c) Explain how missing data were addressed	
		(d) If applicable, explain how loss to follow-up was addressed	
		(e) Describe any sensitivity analyses	
		**Results**	
Participants	13 *	(a) Report numbers of individuals at each stage of study—e.g., numbers potentially eligible, examined for eligibility, confirmed eligible, included in the study, completing follow-up, and analysed	5 Figure 1
		(b) Give reasons for non-participation at each stage	
		(c) Consider use of a flow diagram	
Descriptive data	14 *	(a) Give characteristics of study participants (e.g., demographic, clinical, social) and information on exposures and potential confounders	7, Table 2 Table S1
		(b) Indicate number of participants with missing data for each variable of interest	
		(c) Summarize follow-up time (e.g., average and total amount)	
Outcome data	15 *	Report numbers of outcome events or summary measures over time	Table 3 Figure 1
Main results	16	(a) Give unadjusted estimates and, if applicable, confounder-adjusted estimates and their precision (e.g., 95% confidence interval). Make clear which confounders were adjusted for and why they were included	5,6,7,8,9,10 Table 1 Table 3 Figure 2. Appendix B
		(b) Report category boundaries when continuous variables were categorized	
		(c) If relevant, consider translating estimates of relative risk into absolute risk for a meaningful time period	

Other analyses	17	Report other analyses performed—e.g., analyses of subgroups and interactions, and sensitivity analyses	Supp. Mat S1,S3,S4,S5,S6,S7
		**Discussion**	
Key results	18	Summarize key results with reference to study objectives	10,11,12
Limitations	19	Discuss limitations of the study, taking into account sources of potential bias or imprecision. Discuss both direction and magnitude of any potential bias	13
Interpretation	20	Give a cautious overall interpretation of results considering objectives, limitations, multiplicity of analyses, results from similar studies, and other relevant evidence	10,11,12
Generalisability	21	Discuss the generalisability (external validity) of the study results	12
		**Other information**	
Funding	22	Give the source of funding and the role of the funders for the present study and, if applicable, for the original study on which the present article is based	---

Note: * Give information separately for exposed and unexposed groups. An Explanation and Elaboration article discusses each checklist item and gives methodological background and published examples of transparent reporting. The STROBE checklist is best used in conjunction with this article (freely available on the Web sites of PLoS Medicine at http://www.plosmedicine.org/ (accessed on 15 February 2021), Annals of Internal Medicine at http://www.annals.org/ (accessed on 15 February 2021), and Epidemiology at http://www.epidem.com/ (accessed on 15 February 2021)). Information on the STROBE Initiative is available at http://www.strobe-statement.org (accessed on 15 February 2021).

File S1. CUCEQS© (Questionnaire of theperioperative nursing competencies in patient safety)

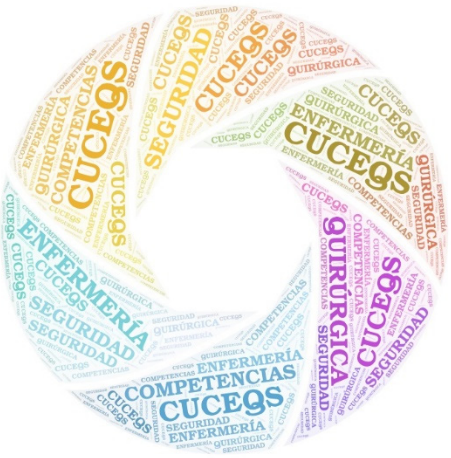

CUCEQS

QUESTIONNAIRE OF THE PERIOPERATIVE NURSING COMPETENCIES IN PATIENT SAFETY

CUCEQS by Ester Peñataro Pintado is licensed under a
Creative Commons Reconocimiento-NoComercial
SinObraDerivada 4.0 Internacional License

CUCEQS QUESTIONNAIRE

Concept:

We present to you the CUCEQS questionnaire. The acronym of this questionnaire, in Spanish, responds to "***CU**ESTIONARIO DE **C**OMPETENCIAS DE LA **E**NFERMERÍA **Q**UIRÚRGICA EN **S**EGURIDAD*". It is a questionnaire designed to assess the competitive level of perioperative nursing focused on surgical patient safety.

To define the term competency is not an easy task, due to the existence of different complementary approaches, but the one that best suits our research is: "Ability to adequately mobilize the set of knowledge, skills and attitudes necessary to carry out diverse activities with a certain level of quality and efficiency". This definition, when talking about quality and (Bisquerra, R. & Pérez, N. (2007). effectiveness, is considered a more advanced and necessary stage of competencies, which is applicable in nurse care, both in the world of universities during the training process of their students, and to nursing professionals when they practice and develop their profession.

This questionnaire focuses on the nursing team and the roles you can develop, depending on the dynamics and needs of the facility you are in, which may be circulating nursing, scrub nursing, anesthesia nursing and postoperative nursing, throughout the perioperative process.

The last term that includes CUCEQS in its acronym is "Safety", also referred to as "Patient Safety" or "Clinical Safety", which involves practicing harm-free health care. This involves developing systems, interventions and activities aimed at reducing the likelihood of system failures and people's errors and verifying what level of competency the professional has acquired.

The error, in healthcare practice, refers to an act of mistakes by healthcare professionals that can contribute to an adverse event. Assuming that being wrong is human is vital to patient safety is the first step in trying to avoid it.

As well as the analysis of personal and system factors that contribute to the error.

The mistake can be analyzed in two ways, the first would be from a perspective focused on the person making the mistake, making her guilty of the happened fact. But you can also perform the analysis from a system-centric perspective, without looking for culprits, but by analyzing errors and learning from them, based on the results obtained. This second perspective is what we will consider in this questionnaire.

Objective:

The objective of the questionnaire is to assess the level of competency perceived by perioperative nursing in surgical patient safety, which will go, weakly acquired to satisfactorily acquired.

Use:

The use of CUCEQS will always be aimed at recognizing both qualities and points of improvement to promote the culture of Surgical Patient Safety.

The questionnaire may be used by the Perioperative Nursing at the end of its training phase, at the end of the Postgraduate Degree in Perioperative Nursing, with the aim of self-assessment and knowledge of both the skills it has acquired and aspects pending improvement.

Likewise, CUCEQS can be used by experienced nurses who wish to evaluate themselves to know the competitive level of defined competencies and identify how they can improve their care practice and apply care plans to safer and more quality surgical patients.

PERIOPERATIVE NURSING COMPETENCY QUESTIONNAIRE IN PATIENT SAFETY

For the preparation of this questionnaire, the competencies of the European Operating Room Nurse Association (EORNA), the standards or recommendations of the National Health System Quality Agency to define The Patient Safety Strategy, have been had as a theoretical reference framework, the qualitative analysis of 4 focal groups carried out, consisting of expert perioperative nurses from 4 public hospitals in the Catalan environment, in which the Safety of the Surgical Patient has been raised and different documents published by the Ministry of Health, Social Services and Equality (Patient Safety Strategy of the National Health System, Practical Guide to Surgical Patient Safety.

This questionnaire defines different domains and perioperative nursing competencies related to Surgical Patient Safety, considering the different roles you can develop anesthesia nurse, scrub nurse, circulating nurse and postoperative nurse throughout the perioperative process, (preoperative, intraoperative and postoperative).

Carefully read and analyze each competition and the concrete actions of each competition.

To answer the questionnaire, consider your overall care experience and be in that most recent situation you have ever experienced.

You should also consider, throughout your professional experience, what nursing roles you have developed, it may be that you have developed one or all the roles, respond depending on this situation to the skills being discussed. This will be considered in the final score of the questionnaire. If you have not been given the opportunity to perform any of the roles in competency 2 related to perioperative care you have the option to answer “Does not apply/0”.

You must answer each item defined for each competition using a Likert Scale from 0 to 4, considering:

NA/0: Not applicable	1: Rarely	2: Sometimes	3: Normally	4: Always

SOCIODEMOGRAPHIC DATA:

IDENTIFICATION CODE. Formed by the last four digits of your ID + the initials of your first and last name:

SEX: Female Male

AGE:

YEARS DEVELOPING THE NURSE PROFESSION:

YEARS DEVELOPING THE NURSE PROFESSION IN THE OPERATING ROOM:

SUBSEQUENT STUDIES:

1. Graduate
2. Master
3. Doctorate
4. Other....

HEALTH CENTER IN WHICH IT DEVELOPS ITS PROFESSIONAL ACTIVITY TODAY:

PERIOPERATIVE NURSE ROLE THAT USUALLY DEVELOPS. CHECK ALL POSSIBLE OPTIONS:

1. Anesthesia Nurse
2. Circulating Nurse
3. Scrub Nurse
4. Postoperative Nurse

COMPETENCY 1: EXERCISES IN ACCORDANCE WITH LEGISLATION, ETHICS AND PROFESSIONAL GUIDANCE WITHIN THE FIELD OF PERIOPERATIVE NURSING

This competency refers to the development of an ethical and legal practice that preserves respect for the patient. This requires knowledge of ethics, regulation, and professional legislation to be developed throughout the perioperative process.

The complexity of the defined competency requires the creation of different levels of competency to, on the one hand, assess whether it is exercised within national and European legislation, professional regulation, and protocols throughout the perioperative process in all its phases (preoperative, intraoperative and postoperative phase). On the other hand, assess whether efforts are directed to provide a safer perioperative care practice, free of avoidable damage and equipped with systems aimed at reducing the likelihood of errors and failures, increasing the likelihood of detecting them if they occur, and mitigating their consequences.

Read and analyze each competency and actions described before answering. You must score each of the actions described according to the role developed within ~~Surgical~~ Perioperative Nursing, according to your professional experience, to ensure the safety of the surgical patient.

You must score on a Likert Scale from 0 to 4.

NA/0: Not applicable; 1: Rarely, 2: Sometimes; 3: Normally; 4: Always.

1.1	**Ability to Meet Safety Standards**
	You present yourself to the patient by name and professional category.
	You actively ask the patient the name, surgery, and laterality, if applicable, to make it part of their own safety, provided that their neurological status allows it. If you are not looking for other patient identification strategies.
	You verify the identification bracelet, medical history, and informed consent.
	Check that the limb or surgical area is marked.
	You ask the patient if they have any known allergies and if they are fasting.
	You check and ask the patient if jewelry, prosthetics, or any metal objects have been removed.
	You justify your above questions to the patient and make it clear that the goal is to ensure the safety of the Surgical Patient.
	You check that you provide all the necessary diagnostic tests according to the surgery.

Highest level of competency: 32.

1.2	**Ability to Apply Surgical Checklist (LVQ) or Surgical Checklist**
	Depending on your dynamics and supporting role, you verbally check the surgical checklist items defined by WHO.
	Depending on your dynamics and supporting role, you record WHO-defined SCI items in real time.
	When you act as a nurse responsible for the time of "Time out"prior to the surgical incision, you lead iteffectively (you claim your time, space to perform it and actively hear from the rest of the surgical team), aloud with all surgical team members present. If the items are not met it will stop.

Highest level of competency: 12.

COMPETENCY 2: PROVIDES PERIOPERATIVE NURSING CARE BY INTEGRATING EVIDENCE-BASED KNOWLEDGE AND PRACTICE INTO A SAFE ENVIRONMENT

Patient safety is one of the concerns of the World Health Organization (WHO), which is why it poses the challenge "Safe Surgery Saves Lives", because although the goal of surgery is to save lives, the lack of safety of surgical professionals can cause serious and irreparable damage that is considered avoidable in at least half of cases. Nursing must respond to the challenge of WHO by European, national and regional institutions. To do this you must acquire the necessary skills and develop holistic and safe nurse care.

NA/0: Not applicable; 1: Rarely, 2: Sometimes; 3: Normally; 4: Always.

2.1	**If you have performed your intervention as an Anesthesia Nurse check the following items in relation to anesthetic technique:**
	You check the administration or administer the antibiotic prescribed between 30–60 min prior to the surgical incision.
	You give a second dose of antibiotic, if necessary, and according to protocol.
	You know and prepare the necessary material for the development of anesthesia.
	You pre-check the proper functioning of the material necessary for the development of the indicated anaesthesia
	Know the medication needed for anesthesia.
	Prepares and identify the medication needed for anesthesia so that it does not lend to any errors.
	You know the sequence to apply to the indicated anesthesia.
	You collaborate correctly in the sequence to apply the indicated anesthesia.
	You demonstrate dexterity and skill in the development of the anesthetic sequence.
	You identify emergency situations and act effectively.
	You recognize its limitations, difficulties and request collaboration if you need it.
	You transfer relevant patient information to the postoperative nurse.

Highest level of competency: 48.

2.2	**If you have performed your intervention as an Anesthesia Nurse or Circulating Nurse check the following items in relation to safety in the patient's placement:**
	You know and collaborate in the safe position indicated according to the surgical intervention.
	You prepare all the necessary resources for the safe placement of the patient according to the surgical intervention.
	You always preserve the privacy, dignity and confidentiality of the patient, (does not leave the patient discovered on the surgical table, does not provide value judgments, only comments on the clinical case with professionals of the surgical team).
	You apply protections at the eye level, on the patient's bone prominences and on the necessary supports according to each surgical position, to avoid alteration of skin integrity.
	Place the necessary fasteners and supports to prevent the patient from falling from the surgical table.
	You lead the time of safe placement of the surgical patient and demand the time needed.

Highest level of competency: 24.

**2.3**	**If you have performed your intervention as an Anesthesia Nurse or Circulating Nurse check the following items in relation to the surgical thermoregulation of the patient:**
	You know the importance of surgical thermoregulation and the complications of surgical hypothermia.
	Apply measures to prevent surgical hypothermia: hot air blankets in the intraoperative phase.
	Apply measures to prevent surgical hypothermia: warm air blankets in the immediate postoperative phase.
	Apply measures to prevent surgical hypothermia: warm air blankets in the preoperative phase.
	Administers hot intravenous fluid therapy.

Highest level of competency: 20.

**2.4**	**If you have performed your intervention as a Circulating Nurse check the following items:**
	You know the material and instruments needed for the indicated surgery or recognize that you have difficulty identifying all the necessary material.
	Prepare the material and instruments needed for the surgery indicated with the Scrub Nurse or recognize that she has difficulty completing all the necessary material.
	Prepare and place the necessary surgical utensils correctly and neatly during surgery.
	You work in an orderly manner in the operating room during surgery.
	You perform and record the gauze and instrumental count with the Scrub Nurse before starting the surgery.
	You perform and record the gauze and instrumental count with the Scrub Nurse during surgery.
	You perform and record the gauze and instrumental count with the Scrub Nurse before suturing the surgical incision.
	You provide the necessary surgical material and instruments, respecting infertility strictly, at all times, throughout the indicated surgery.
	You adapt and respond effectively to a new and unexpected situation throughout the surgery.
	You recognize your limitations, difficulties, and request collaboration if you need it.
	You control and avoid the circuit of people in the operating room.
	Avoid unnecessary entry or exit from surgical team members.
	You handle and preserve, appropriately, the biological samples that are delivered to you by the Scrub Nurse.
	You transfer relevant patient information to the postoperative nurse.

Highest level of competency: 56.

**2.5**	**If you have performed your intervention as an Scrub Nurse check the following items:**
	You ask and you are interested in the surgery that's going to develop.
	You know the material and instruments needed for the surgery indicated next to the circulating nurse.
	You prepare the necessary material and instrument for the surgery indicated next to the circulating nurse.
	You recognize its limitations, difficulties, and request collaboration if you need it.
	Perform surgical hand hygiene according to strict asepsis regulations.
	You put on your own surgical attire follows strict sterile regulations.
	You collaborate in the placement of surgical attire of the rest of the surgical team following strict sterile regulations.
	You handle surgical instruments during surgery following strict maintenance of sterile regulations.
	You perform gauze and instrumentation connection with the circulating nurse prior to the surgical incision.
	You perform gauze and instrumental connection with the circulating nurse during the surgery.
	You perform gauze and instrumentation connection with the circulating nurse before suturing the surgical incision.
	You adapt and respond effectively to a new and unexpected situation.
	You anticipate the resources needed during the development of surgery.
	You collect and preserve the biological samples obtained adequately.
	You connect the surgical drains sterilely and then they are fixed.
	You place the dressing on the surgical wound and the drains in a sterile way.

Highest level of competency: 64.

2.6	**If you have performed your intervention as an Anesthesia Nurse, Circulating Nurse, or Scrub Nurse in relation to the use of the electric scalpel check the following items:**
	You know the safe rules for the use of the electric scalpel.
	You apply the safe rules for the use of the electric scalpel.
	You know the dispersion plate placement areas according to the surgery indicated.
	You check the integrity and condition of the skin where to attach and place the scatter plate to ensure direct contact with the patient's skin.
	You regulate the parameters of the electric scalpel according to the surgery and the patient.
	Knowhow to prevent and act on flammability, flash or surgical fire.

Highest level of competency: 24.

2.7	**If you have performed your intervention as a Postoperative Nurse check the following items:**
	You report on the surgical interventions that develop and the characteristics of the patients you are going to care for in the post-surgical recovery unit.
	Prepare the necessary material and resources in the post-surgical recovery unit before the arrival of patients.
	You check the proper functioning of the material and available resources.
	Prepare, label, and administer the necessary medication and analgesia upon patient arrival.
	You receive information about the development of anesthesia and surgery performed to the patient who transmits the anesthesia nurse or anesthesiologist.
	You identify the patient using the identification bracelet.
	You verify that the documentation and medical history that accompanies the patient is correct.
	Monitor vital signs and value the patient's hemodynamic status upon arrival at the post-surgical recovery unit.
	You monitor and record vital signs every 15 min or less, depending on the patient's condition.
	You control the patient's airway in their post-surgical recovery process.
	You value the patient's state of consciousness upon arrival at the recovering unit.
	You identify the devices established in the patient (probes, catheters, drains...) and their proper functioning.
	You check the condition of the dressing upon arrival at the recovering unit.
	You check the patient's allergies.
	Define the right care plan for each patient.
	You successfully administer treatment by the anesthesiologist or surgeon.
	You value the hemodynamic evolution of the patient in the recovering unit.
	You apply heat blanket or other available resources to conserve the patient's temperature during their stay in recovering unit.
	You establish effective communication with the patient, adapting to the patient's condition.
	You establish an empathetic relationship with the patient in their post-surgical recovery process.
	Inform the patient of the planned care plan.
	You record all activities or interventions performed in the postoperative recovering of the patient.
	You recognize your limitations, difficulties and request collaboration if you need it.

Highest level of competency: 92.

2.8	**If you have performed your intervention as an Anesthesia Nurse, Circulanting Nurse or Postoperative Nurse in relation to pain check the following items:**
	You ask the patient if they experience pain and locate them.
	You use the EVA scale or another to assess the pain of the patient.
	You know the resources available to relieve pain.
	Plan the activities and resources available to relieve pain.
	Apply the activities and resources available to relieve pain.
	You evaluate with the patient, using the EVA scale or another, the effectiveness of the resources applied to the patient and share it with the interdisciplinary team.
	You can propose the need to modify the resources applied if necessary.

Highest level of competency: 28.

COMPETENCY 3: ESTABLISHING AND KEEPING EFFECTIVE INTERPERSONAL RELATIONSHIPS WITH PATIENTS AND SURGICAL EQUIPMENT DURING THE PERIOPERATORY PROCESS

Due to the complexity of the surgical process and the participation in it of a multidisciplinary team it is necessary to assess communication with the team, with patients and their link with the safety of the surgical patient.

The European Quality Model or EFQM of Excellence can be applied to health management. This is a total quality model that bases its development on self-assessment continuously and that considers leadership as a key factor, through which results are achieved, emphasizing activities that give value and eliminating those that do not add value. Therefore leadership skills have been included.

NA/0: Not applicable; 1: Rarely, 2: Sometimes; 3: Normally; 4: Always.

3.1	**Ability to establish effective communication with the patient during the perioperative process**
	You can maintain smooth and effective communication with the patient.
	You are able to identify the emotional state of the patient and establish an empathetic attitude.
	You are able to adapt the information to the patient's profile, language and understanding.
	You are able to establish a relationship of trust with the patient.
	You are able to solve patient doubts at every stage of the perioperative process.
	You are able to claim the time needed to establish effective and effective communication with the patient.
	You are able to handle conflicting situations of communication with the patient effectively.

Highest level of competency: 28.

3.2	**Ability to develop strategies to promote communication and teamwork**
	You are able to maintain smooth and effective communication with the perioperative nursing team to know the patient's needs during the surgical process.
	You are able to maintain assertive communication with the perioperative nursing team to jointly define the nurse care plan during the surgical process.
	You are able to maintain smooth and effective communication with the rest of the surgical team to know the patient's needs during the surgical process.
	You are able to maintain assertive communication with the rest of the surgical team to jointly define the care plan at the interdisciplinary level during the surgical process.
	Transfer relevant patient information to postoperative nurse.
	You are able to handle conflicting situations of communication with the surgical team effectively.
	You are able to claim the time needed to define priorities related to patient safety and establish fluid communication with the surgical team.

Highest level of competency: 28.

3.3	**Nursing leadership capacity**
	You are accepted by the interdisciplinary team to lead the surgical process.
	You are accepted by the interdisciplinary team to lead the surgical process.
	You are able to create group cohesion in the interdisciplinary team.
	You are able to create group cohesion among all team members.
	You are able to define the guidelines for a safe and quality perioperative process, in an orderly and chronological manner.
	The interdisciplinary team follows the perioperative process guidelines defined by the leader in the right direction.
	The interdisciplinary team follows the perioperative process guidelines defined by the leader in the right direction.
	You are considered /or a reference to ask questions and raise doubts.
	You are considered a reference to train novice nurses who join the service.
	You tolerate stressful situations by showing self-confidence and nurse interventions.
	You search and share with the team the best scientific evidence about the safe care plan to develop.

	You are able to transmit to the team an impulse of permanent action, construction and positive energy in relation to patient safety.
	You are able to give the team confidence in their own challenges.
	You are able to show that the achievements are of the team and are not individual.
	You are able to recognize their limitations, conflicts and, look for alternatives/solutions.

Highest level of competency: 60.

COMPETENCY 4: PROMOTING THE SAFETY CULTURE OF THE SURGICAL PATIENT

The safety culture is the result of values, attitudes and behaviors that characterize the functioning of the surgical team with the priority of achieving the prevention of the appearance of adverse effects related to health care.

The importance of safety culture recognizes access to quality healthcare and that continuous improvement of quality of care is a key goal for surgical patient safety in our study.

NA/0: Not applicable; 1: Rarely, 2: Sometimes; 3: Normally; 4: Always.

**4.1**	**Capacity to foster a professional-centered patient safety culture**
	Analyses and reflections on the care process at the end of the day.
	You are able to design your own care quality indicators throughout the perioperative process.
	You are able to share, at the intradisciplinary level, your own indicators of care quality of the entire perioperative process.
	You are able to share, at the interdisciplinary level, your own indicators of care quality of the entire perioperative process.
	You are able to propose activities to improve the safety of the surgical patient.

Highest level of competency: 20.

**4.2**	**Promotion of patient safety culture centered in professional with respect to organization or institution (linked to basic quality standards and surgical process evaluation tools in all its phases)**
	You are interested in whether there’s a security culture on the part of the organization.
	You show interest in how security culture is applied in the organization/institution.
	Interested in forming in safety culture and surgical risk management within an organization/institution.
	You are interested in the quality indicators of the institution/organization related to patient safety in the perioperative process.
	You propose actions to improve the safety culture in the perioperative process in the institution/organization.
	You encourage learning from adverse incidents and events as opposed to guilt or punishment.

Highest level of competency: 24.

**4.3**	**Ability to identify, notify and communicate errors**
	You identify your mistake.
	Inform the rest of the team of your mistake.
	You report your mistake through the circuit described in your workplace
	You apply measures to lessen the side effects of the error.
	You analyze your mistake and its causes.
	You perceive error as an apprenticeship and not as punitive.
	You design and propose procedural improvements that can avoid error.
	If you identified another team member’s error, you’d let them know
	If the error is of importance to the patient, you would report it to the team.
	If the error is of significance to the patient shows interest in whether they have been informed
	You report another team member’s error according to the center’s procedure.
	Encourage error analysis with the rest of the surgical team.
	You encourage discussion of the error in team meetings.
	You encourage the creation of a working group for error logging and analysis.

Highest level of competency: 56.

4.4	**Nurse's ability to develop the scientific knowledge of every moment of the perioperative process**
	Recognize your knowledge or skills limitations.
	You are actively committed to your training.
	You ask for collaboration with expert professionals in the field to solve your doubts.
	You're interested and know the protocols and clinical guidelines available depending on the surgical intervention.
	You're committed to being up to date on everything about the surgical process.
	You know the scientific evidence of the resources available to plan your safe nurse interventions.
	You apply the best scientific evidence in your nurse interventions throughout the perioperative process.
	You apply the protocols defined by the institution according to the surgery.
	You encourage the need to update protocols on a regular and consensual basis by surgical team members.
	You're looking for safe alternatives when you don't have the optimal resources to follow the protocol defined by surgery or stop the procedure if it's not safe.

Highest level of competency: 40.
